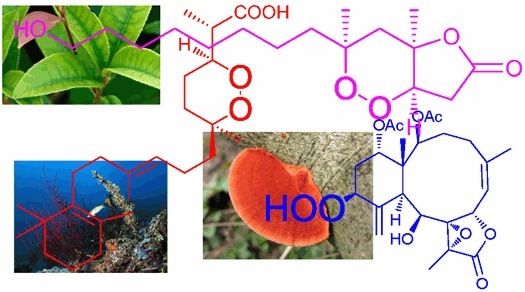# Peroxy natural products

**DOI:** 10.1007/s13659-013-0042-7

**Published:** 2013-09-06

**Authors:** Dong-Ze Liu, Ji-Kai Liu

**Affiliations:** 1 Tianjin Institute of Industrial Biotechnology, Chinese Academy of Science, Tianjin, 300308 China; 2 State Key Laboratory of Phytochemistry and Plant Resources in West China, Kunming Institute of Botany, Chinese Academy of Sciences, Kunming, 650201 China

## Abstract

This review covers the structures and biological activities of peroxy natural products from a wide variety of terrestrial fungi, higher plants, and marine organisms. Syntheses that confirm or revise structures or stereochemistries have also been included, and 406 references are cited.

**1 Introduction**
**2 Marine Metabolites**
**2.1 1,2-Dioxane Carboxylates**
**2.2 1,2-Dioxolane Carboxylates**
**2.3 Fatty Acid Derived Peroxy Ketals**
**2.4 Diterpenes**
**2.5 Other Marine Metabolites**
**3 Terrestrial Sources**
**3.1 Monoterpenes**
**3.2 Sesquiterpenes**
**3.2.1 Guaianes**
**3.2.2 Eudesmanes**
**3.2.3 Bisabolanes and Germacranes**
**3.2.4 Sesquiterpene Dimers**
**3.2.5 Other Sesquiterpenes**
**3.3 Diterpenes**
**3.4 Triterpenes**
**3.5 Others**
**4 Steroidal Peroxides**
**5 Fatty Acid Metabolites**
**6 Conclusions**
**7 Acknowledgements**

## 1 Introduction

This review, which is of the literature from 1998 to 2013, follows the pattern of its predecessors and is devoted to the new occurrence of peroxy compounds[1,2] and described 639 naturally occurring peroxides from 406 articles. In the past more than 10 years, peroxy compounds have been isolated from a wide variety of terrestrial fungi, higher plants, and marine organisms, especially sponge species, many of which exhibited diverse biological properties such as anti-inflammatory, antimalarial, antimicrobial, cytotoxic, antitumor activities, and so on.

As a result of the potential for new drug discovery, peroxy compounds have attracted the attention of biologists and chemists throughout the world. So far, some reviews have dealt with the class of natural peroxides: "Peroxy natural products",[1,2] "Natural peroxy anticancer agents",[3] "Bioactive peroxides as potential therapeutic agents",[4] and "Naturally occurring peroxides with biological activities".[5] Other general reviews are: "Monoterpenoids", "Sesquiterpenoids", "Diterpenoids", "Sesterterpenoids", "Triterpenoids", and "Marine natural products" all published in the journal *Natural Product Reports* covering from 1998 to 2011. References to other reviews are appropriately placed in the following sections.

In this review, we showed the structures of new peroxides, and previously-reported ones where there has been a structural revision or a newly-established stereochemistry. Previously-reported peroxides for which first syntheses or new bioactivities are described are referenced, but separate structures are generally not shown. Relevant data published in MEDLINE, Google Scholar, and SciFinder since 1998 have been gathered to formulate the following review.

## 2 Marine Metabolites

**2.1 1,2-Dioxane Carboxylates:** Marine sponges, notably those from the genera *Plakortis* and *Plakinastrella,* continued to provide a source for six-membered ring cyclic peroxides that incorporate a lactone ring. Plakortolides K–S (**1–9**)[7] were isolated from the Australian sponge *Plakinastrella clathrata.* Detailed configurational investigation also revealed that the structure for previously reported plakortolide E[6] should be

*To whom correspondence should be addressed. E-mail: liu_dz@tib.cas.cn (D.Z. Liu); jkliu@mail.kib.ac.cn (J.K. Liu)

revised to a non-peroxidic metabolite and the commonly assumed biosynthesis of the cyclic peroxide *via* Diels-Alder addition of singlet oxygen is incorrect.[7] The first total synthesis of *seco*-plakortolide E also supported the structural revision of plakortolide E.[8]

Continuing investigation of the same sponge, *P. clathrata*, afforded an additional set of 16 plakortolide metabolites **10**–**25**.[9] A Jamaican collection of *Plakinastrella onkodes* yielded two cyclic peroxides, plakortolide F **26** and plakortolide G **27**. The absolute stereochemistry of plakortolide G was proposed from a combination of optical rotation and molecular modelling data. Plakortolide G exhibited potent inhibitory activity against the AIDS opportunistic parasitic infection *Toxoplasma gondii*.[10] The trivial name plakortolide F was also given to a different peroxide **28**, which was obtained from an unidentified species of *Plakinastrella* collected in the Seychelles.[11] Two 1,2-dioxane peroxylactones, plakortolides H and I **29** and **30**, have been isolated from a Madagascar specimen of *Plakortis* aff *simplex,* both of which were cytotoxic against a range of human tumour cell lines.[12] Several years later, the relative and absolute configurations of plakortolide I were revised on the basis of synthetic studies and reassignment of the NMR data,[8,13] thereby establishing that the metabolite isolated was *ent*-plakortolide I **31**. Whilst the trivial name plakortolide I has been proposed for an unnamed plakortolide metabolite **32** from the Philippine Sponge *Plakinastrella* sp., whose absolute stereochemistry was determined by application of Mosher's method to a derivative.[14] The authors also detail the unreliability of specific rotation measurements in the determination of absolute

**1** R = H
**2** R = OH

**3** R = H
**4** R = OH

**5**

**6**

**7**

**8**

configuration within the plakortolide class of metabolites in the same paper.[13] The first total synthesis of **32** has been achieved using a [2 + 4] photocycloaddition of a singlet oxygen to a diene and iodolactonization as key steps.[15] A different species of *Plakortis*, *P. halichondrioides*, yielded additional peroxide-lactone named plakortolide J **33**, the absolute stereostructure of which was determined by degradation reactions followed by application of Kishi's method for the assignment of absolute configuration of alcohols.[16] Synthetic efforts in construction the 1,2-dioxane ring of plakortolides have been described.[17,18]

A further cyclic peroxide **34**, with a terminal phenyl group but lacking the lactone, was isolated from *P. Clathrata*.[9] The ester represents further structural variation within the growing family of cyclic peroxy sponge metabolites.

The stolonoxides and stolonic acids are a family of natural aliphatic endoperoxides obtained from the samples of marine ascidians belonging to the genus *Stolonica.* Stolonoxide A **35**, the first member of the series, was isolated as its methyl ester from the marine tunicate *Stolonica socialis*.[19] A further investigation conducted on the same species yielded stolonoxides B–D **36**–**38**, with strong cytotoxic activity against a panel of five tumor cell lines.[20] The methyl ester derivatives of stolonoxides A and C have been identified as potent inhibitors of the mitochondrial respiratory.[21] In addition, two new members of this structural class possessing a longer aliphatic chain, stolonic acids A and B **39** and **40**, were isolated from an Indian Ocean Ascidian *Stolonica* species. Both compounds exhibited antiproliferative activity against selected human melanoma and ovarian tumor cell lines, with $IC_{50}$

Dong-Ze Liu was born in Shandong province, China, in 1976. He received his B.Sc. (2001) and M.Sc. (2004) degrees from Qingdao Agricultural University and Shanghai Jiao Tong University, respectively. In 2007, he completed his Ph.D. degree in Phytochemistry from Kunming Institute of Botany, Chinese Academy of Sciences (CAS). From 2008 to 2010 he worked as a postdoctoral fellow at the South China Sea Institute of Oceanology, CAS, in Marine Natural Products. In 2011, he joined the Tianjin Institute of Industrial Biotechnology, CAS. His current research interests are focused on the isolation, structure determination, and structural modification of natural products.

Ji-Kai Liu is a professor of Kunming Institute of Botany since 1997. He acquired his Ph.D. degree at Lanzhou University in 1988. From 1993 to 1994 he worked as a research fellow of Alexander von Humboldt at the University of the Saarland in Germany. Then he worked as a research scientist at the Pharma Research Center of Bayer AG in Germany. His field of interest concerns natural bioactive compounds from higher fungi. He has published over 200 peer-reviewed articles in international journals including *Chem. Rev., Angew. Chem. Int. Ed., Nat. Prod. Rep., and Org. Lett.* etc. He is the author of the book Mycochemistry and also one of the inventers for ten patents. He is the main founder of *BioBioPha Co. Ltd.* and the open access journal *Nat. Prod. & Bioprospect.*.

**9**

**10**

**11**

**12**

**13**

**14**

**15**

**16**

**17**

**18**

**19**

**20**

**21**

**22**

**23**

**24**

**25**

**26**

values of approximately 0.05–0.1 μg/mL.[22] Two new members of the stolonoxide family, stolonoxides E and F **41** and **42**, were obtained from samples of the marine ascidian *S. socialis.* Both compounds displayed low micromolar cytotoxicity against a panel of human tumor cell lines.[23]

The marine sponges of the genus *Plakortis* are also prolific producers of cyclic polyketide peroxides and structurally related compounds that exhibit a broad spectrum of biological properties. The bioactive cyclic peroxide plakortide Q **43** has been isolated from marine sponge *P. zyggompha*, together with six cyclic peroxide analogues **44**–**49** in their methyl ester forms. The relative stereochemistry of the 1,2-dioxane ring was established after interpretation of the coupling constant values and the NOESY data.[24] Interestingly, a sample of the crude extract of the sponge left standing in methanol for one year yielded the methyl esters directly; this finding may go some way to accounting for the prevalence of methyl esters as reported metabolites of *Plakortis* species. The name plakortide Q was also proposed for a different peroxide **50**, which was isolated from the Caribbean sponge *P. Simplex.*[25] In the same paper, the complete spectroscopic and stereostructural assignments of known 3-*epi*-plakortin has been reported. Three further cyclic peroxides, dihydroplakortin **51**, plakortides I **52** and J **53**, were obtained from the same source, *P. Simplex,* by the same group, as well as providing the

**27**

**28**

**29**

**30**

**31**

**32**

**33**

**34**

**35**

**36**

**37**

**38**

absolute stereochemistries of known plakortin and plakortide H.[26,27] The first synthesis of dihydroplakortin **51** has been achieved, featuring a one-pot three-step hydroperoxysilylation/cyclization reaction for the construction of the endoperoxide ring system.[28] An insight into the mechanism of the antimalarial action of plakortin and dihydroplakortin, simple 1,2-dioxanes isolated from the sponge *P. Simplex,* has been reported.[29]

**39**

**40**

**41**

**42**

The Australian marine sponge *Plakortis* sp. yielded two plakortide Q derivatives **54** and **55**. Both were potent (nM) inhibitors of *Trypanosoma brucei.*[30] Six cyclic peroxides **56–61** were isolated from an Okinawan *Plakortis* sp. and one of these, the peroxide **61**, was shown to be cytotoxic.[31,32] The antileishmanial peroxides **62** and **63** were reported from *P.* aff. *angulospiculatus* collected from Palau together with peroxide **64**, which were inactive.[33] Peroxides **56** and **64** have the same gross structure but the difference in optical rotations suggests that they have different stereochemistries. Fractionation of the sponge *Plakortis* sp. collected around the Amirantes Islands provided peroxides **63**, **65** and **66**.[34] The relative and absolute stereochemistry of the cyclic peroxide **67**, originally isolated from *P. angulospiculatus*,[35] has been proposed by comparison to the optical rotation and NMR spectral data of synthesized diastereomers.[36]

Two independent collections of an undescribed sponge *Plakortis* sp. from Discovery Bay, Jamaica, yielded four cyclic peroxides plakortides I–L **68**–**71**, and two related compounds **72** and **73**, respectively.[37,38] Plakortide I represents the first report of a polyketide-derived cyclic peroxide with an *α,β*-unsaturated ketone moiety in the side chain and exhibits significant antimalarial activity against the W2 Clone of *Plasmodium falciparum* with an $IC_{50}$ value of 570 ng/mL, whilst both **72** and **73** exhibited significant antimicrobial

**43**

**44**

**45**

**46**

**47**

**48**

**49**

**50**

**51**

**52**

**53**

**54**

**55**

**56**

**57**

**58**

**59**

**60**

activity against pathogenic bacteria and fungi with $IC_{50}$ values of 0.9–5.0 μg/mL and 0.7–8.0 μg/mL, respectively. The plakortides named I and J have been renamed plakortides M and N as the trivial names had been used previously for related metabolites isolated from *P. Simplex.*[27] Unfortunately, the trivial names plakortides M and N were also proposed for another two compounds **74** and **75** from the Caribbean marine sponge *P. Halichondrioides*, which exhibited potent cytotoxicity to an array of human tumour cell lines.[39] A Japanese specimen of *Monotria japonica* yielded the monotriajaponides B–D **76–78**, which can lyse starfish oocytes without disruption of nuclear structure.[40] Interestingly, the absolute stereochemistries of **76–78**, as determined by reduction and a modified Mosher method, were opposite to those determined for the plakortides **74** and **75**. Investigation of the bioactive crude extract from the sponge *P. angulospiculatus* from Brazil led to the isolation of the cyclic peroxide plakortenone **79**.[41] A sample of the Norwegian sponge *P. simplex* was found to contain two cyclic peroxides **80** and **81**, of which **81** exhibited moderate *in vitro* activity against several solid human tumor cell lines with $IC_{50}$ values in the range 7–15 μg/mL.[42] An Indonesian sponge *P. nigra* was the source of two isomeric cytotoxic *trans* epoxides, plakorstatins 1 (**82**) and 2 (**83**).[43]

Three cytotoxic cyclic peroxides, ethyl plakortide Z **84**, ethyl didehydroplakortide Z **85**, which demonstrated selective activity *in vitro* against solid tumors but lacked activity *in vivo*, and methyl didehydroplakortide Z **86** were isolated from *P. lita* collected from Papua New Guinea.[44] An Okinawan specimen of the same species provided two futher cytotoxic endoperoxides, haterumadioxins A and B **87** and **88** with moderate cytotoxicity.[45] Plakortide F, originally isolated from *P. Halichondrioides*,[46] interfered with $Ca^{2+}$ homeostasis to mediate the antifungal activity.[47]

**61**

**62** R = H
**63** R = $CH_3$
**65** R = $C_2H_5$

**64**

**66**

**67**

**68**

**69**

**70**

**71**

**72**

**73**

**74**

**75**

**76** $R_1 = CH_3$, $R_2 = H$
**77** $R_1 = CH_3$ $R_2 = CH_3$
**78** $R_1 = Et$, $R_2 = CH_3$

**79**

**80**

**81**

**82** 9*S*, 10*S* or 9*R*, 10*R*
**83** 9*R*, 10*R* or 9*S*, 10*S*

**84**

**85** R = $CH_2CH_3$
**86** R = $CH_3$

**87**

**88**

**89**

A Jamaican collection of *P. Halichondrioides* afforded a peroxide acid **89** with moderate antifungal activity.[48] A two-sponge complex comprising *P. halichondrioides* and *Xestospongia deweerdtae* (Bahamas) yielded one *ω*-phenyl polyketide peroxide named plakinic acid K **90**. The absolute configurations of the isolated chiral centres were determined using liposomal circular dichroism and comparison with synthetic standards.[49]

**90**

**91** R = H
**92** R = Me

**93**

**94**

**95**

**96**

**97**

**98**

**99**

**100**

Fractionation of the *P. onkodes* extract led to the isolation of the cytotoxic cyclic peroxide methyl capucinoate A **91** and the previously reported, but incompletely characterized, aromatic peroxide **92**.[50] Since *P. onkodes* was extracted in MeOH, the methyl esters **91** and **92** may be isolation artifacts.[51] Four aromatic peroxides **93–96** were isolated from *Plakortis* sp. (Orote Peninsula, Guam), of which compounds **93** and **96** showed weak activity against *Staphylococcus aureus*, with MIC values of 128 and 64 μg/mL, respectively.[52] Plakinic acid I **97** was obtained from *P. Halichondrioides*, and the absolute configuration determined from CD curves by degradation and liposomal ordering of naphthamide derivatives.[53] Methylation of the crude extract of a *Sigmosceptrella* sp. from Southern Australia with diazomethane produced a mixture of products, from which nuapapuin methyl ester **98** and sigmosceptrellin D and E methyl esters **99** and **100** were isolated and identified. Their relative stereochemistries were assigned by established empirical rules and absolute stereochemistries by the advanced Mosher procedure. A plausible biosynthetic pathway has also been proposed that rationalizes key transformations in the biosynthesis of known norterpene cyclic peroxides and related norterpene ketones, dienes and sigmosceptrins.[54]

Sponges of the genus *Diacarnus* are known to produce terpene peroxides and related metabolites. A norsesterterpene acid, named muqubilone **101**, was isolated from the Red Sea sponge *D. Erythraeanus*. It showed *in vitro* antiviral activity against herpes simplex type 1 (HSV-1).[55] The same compound **101**, named aikupikoxide A, was also isolated almost at the same time by the Scheuer group from the lipophilic extract of the Red Sea sponge *D. Erythraenus* along with three other cytotoxic cyclic norterpene peroxides, aikupikoxides B–D **102–104**.[56] The same source, *D. Erythraenus,* afforded another three cytotoxic norsesterterpenoid peroxides, tasnemoxides A–C **105–107**.[57]

Bioassay-guided isolation of *D. Levii* collected from Papua New Guinea led to the isolation of four norsesterterpene peroxides, diacarnoxides A–D **108–111**, with diacarnoxides A and B displaying cytotoxic properties and increased activity under hypoxic conditions.[58] Chemical investigation of the sponge *D. megaspinorhabdosa* provided a series of norterpene

Springer

**101**

**102**

**103**

**104**

**105**

**106**

**107**

derivatives, diacarperoxides A–G **112**–**118**, of which, diacarperoxide D was cytotoxic.[59] Re-investigation of *D. megaspinorhabdosa* afforded one further norsesterpene cyclic peroxide, diacarperoxide S **119**, which exhibited strong cytotoxic and antimicrobial activities.[60]

Examination of *D. bismarckensis* (Sanaroa, Papua New Guinea) led to the isolation of two peroxiterpenes *ent*-(–)-muqubilone **120** and (+)-muqubilone B **121**, active against *Trypanosoma brucei* (African sleeping sickness).[61] Specimens of *D.* cf. *spinopoculum* from the Solomon Islands and Papua New Guinea yielded a series of norterpenes including four norsesterterpene peroxides, *ent*-muqubilin A **122**, *ent*-epimuqubilin A **123**, muqubilin B **124**, and epimuqubilin B **125**, and two norditerpene peroxides, nuapapuin B **126** and epinuapapuin B **127**, all of which were evaluated for cytotoxicity using a soft agar assay system and the NCI's 60 cell-line screening. Overall, the norsesterterpene peroxides were less selective as cytotoxins than norditerpene peroxide analogs.[62] The norsesterterpenoid peroxide, *epi*-muqubilin A **122**, inhibited nitric oxide (NO) production in lipopolysaccharide (LPS)-activated murine macrophage RAW 264.7 cells,[63] and suppressed cyclooxygenase-2 *via* IKK/IκB/NF-κB pathways.[64,65] Esterification of carboxylic acid mixtures from the New Caledonian sponge *D. levii* resulted in the isolation of the benzyl esters of *ent*-muqubilin A **122** and deoxydiacarnoate B **128** and the methyl ester of diacarnoate B **129**, all of which were screened for antimalarial activity.[66]

Examination of the Taiwanese sponge *Negombata cortica* revealed a series of related peroxide terpenoids negombatoperoxides A–D **130**–**133**.[67] Three norsesterterpene cyclic peroxides named trunculins G–I **134**–**136** were isolated as their methyl esters from an Australian *Latrunculia* sp., whose absolute stereochemistry about the cyclic peroxide terminus was established by application of the Horeau and Mosher procedures.[68]

**108**

**109**

**110**

**111**

**112**

**113**

**114**

Investigation of a southern Australian sponge of the genus *Mycale* resulted in the isolation of one norsesterterpene mycaperoxide G methyl ester **137**, which was obtained after treatment of the crude extract with diazomethane.[69] The absolute stereochemistry previously assigned to mycaperoxide F methyl ester by application of the Horeau procedure has been revised by application of the Mosher procedure in the same paper. Bioassay-guided isolation of a Thai marine sponge *Mycale* sp. afforded a cytotoxic norsesterterpene peroxide mycaperoxide H **138**. Its relative and absolute stereochemistries were established by standard methodology, including chemical interconversions.[70] Synthetic efforts towards mycaperoxide B, originally isolated from a *Mycale* sp. from Thailand[71], have been reported using a biomimetic approach.[72,73]

**115**

**116**

**117**

**118**

**119**

**2.2 1,2-Dioxolane Carboxylates:** Although the majority of cyclic peroxides contain 1,2-dioxanes, while a growing number possess the more rare 1,2-dioxolane ring system. Bioassay-guided purification of a *Plakinastrella* species collected in the Seychelles led to the isolation of two moderately antifungal plakinic acid F **139** and epiplakinic acid F **140**, containing a conjugated triene on the side chain.[11] Examination of a Puerto Rican collection of *Plakortis halichondrioides* resulted in the isolation of two polyketide endoperoxides, epiplakinic acid F methyl ester **141** and epiplakinidioic acid **142** as well as providing the absolute configuration of known epiplakinic acid F.[16] The antifungal plakortisinic acid **143** was isolated from a species of Jamaican *Plakortis*. The absolute configuration was determined by comparison of calculated and experimental optical rotations.[74]

A Madagascar specimen of *P.* aff. *simplex* yielded one cyclic peroxide, andavadoic acid **144**, which was cytotoxic against a range of human tumour cell lines.[13] Two peroxide acids **145** and **146**, isolated from *P. onkodes* collected in Florida, possessed moderate antifungal activity.[48] The Palauan Sponge *P. nigra* provided two cyclic peroxides designated epiplakinic acids G and H **147** and **148**. Isolated metabolites were found to inhibit the growth of HCT-116 cells.[75] The first asymmetric synthesis of 1,2-dioxolane-3-acetic acids has been reported, and a further optimized strategy was applied to the synthesis of four stereoisomers of plakinic acid A,[76] allowing a complete configurational assignment of plakinic acid A.[77]

One $\omega$-phenyl polyketide peroxide, plakinic acid L **149**, was isolated from a two-sponge association of *P. halichondroides* and *X. deweerdtae*.[49] Synthesis of four possible diastereomers of plakortide E[78] established the absolute configuration of plakortide E as shown.[79] Plakinic acid J **150** was obtained from *P. Halichondrioides*, and the absolute configuration determined from CD curves by degradation and liposomal ordering of naphthamide derivatives.[53] The Philippine sponge *Plakinastrella* sp. yielded two further cyclic peroxides **151** and **152**.[12]

**2.3 Fatty Acid Derived Peroxy Ketals:** Two acetylenic cycloperoxides named peroxyacarnoic acids C and D (**153** and **154)** have been isolated as their methyl esters from the Indian sponge *Acarnus bicladotylota*,[80] and the structurally related methyl peroxyacarnoates A and B **155** and **156**, have been found from the Red Sea marine sponge *A.* cf. *bergquistae*.[81] The absolute stereochemistries of **153–155** were determined by the application of Mosher's method. The syntheses of methyl peroxyacarnoates A and D have been accomplished on the basis of chemoselective ozonolysis within a polyunsaturated framework and Pd-mediated cross-couplings of a functionalized 1,2-dioxane.[82] The endoperoxyketal polyketides manadoperoxides A–D **157–160** with moderate antimalarial activity were isolated from the Indonesian sponge *Plakortis* cfr. *simplex* and their stereostructures were established by means of spectroscopic data and semisynthetic transformations.[83]

Chemical investigation of the marine sponge *P.* cfr. *lita* afforded a library of endoperoxyketal polyketides named manadoperoxides E–K **161–167** and peroxyplakoric ester C **168**, of which manadoperoxides F **162**, H **164**, I **165**, and K **167** exhibited remarkable antitrypanosomal activity without cytotoxicity. The report have also demonstrated unambiguously that the endoperoxy group does not confer *per se* activity against Trypanosoma.[84] The structures and absolute stereochemistries of known natural products chondrillin[85] and its C-3 epimer, plakorin[86,87] have been confirmed by syntheses of (+)- and (–)-chondrillin and (+)-and (–)-plakorin.[88]

**2.4 Diterpenes:** One eunicellin-type diterpenoid astrogorgin G **169** has been repored from a Chinese gorgonian *Astrogorgia* sp.,[89] and the structurally related oxylitophynol **170** and klysimplexin D **171** have been obtained from the soft coral *Cladiella krempfi* and *Klyxum simplex*, respectively.[90,91] From a biogenetical standpoint, oxylitophynol might derive from the formal photo-oxygenation of the corresponding $\Delta^{6,7}$ olefin. Another two substances of this type, briarellin K hydroperoxide **172** and briarellin D hydroperoxide **173**, have been isolated

120

121

122

123

124

125

126

127

128 R = $H_2$
129 R = O

130

131

132

133

134

135

136

137

138

from a Puerto Rican collection of *Briareum polyanthes*,[92] and this study has also led to a revision of the structure of previously reported briarellin A[93] to **174**. The structure originally assigned to 11-acetoxy-4-deacetoxyasbestinin F[94] has been revised to **175**.[95] Spectroscopic discrepancies observed for the enantioselectively synthesised structure originally proposed for alcyonin[96] have led to the proposal that the correct structure of the natural product is the allylic peroxide **176**.[97]

Two dolabellane diterpenoids **177** and **178** with anti-protozoan activity have been obtained from a Colombian gorgonian coral of the genus *Eunicea*.[98] New diterpenoid, **179** having a dolabellane skeleton, was isolated from the Okinawan soft coral of the genus *Clavularia*. This diterpenoid showed cytotoxic activity against several tumor cell lines.[99] Other compounds of this type included calyculatine **180** from *E. calyculata*, and (1*R**,7*R**)-7-hydroperoxydolabella-

**139**

**140**

**141**

**142**

**143**

**144**

**145**

**146**

**147**

**148**

**149**

**150**

**151** R = $CH_3$
**152** R = H

**153**

**154**

**155**

**156**

**157** R = $CH_3$
**158** R = $CH_2CH_3$

**159**

**160**

4(16),8(17),11(12)-triene-3,13-dione **181** from *C. inflata*.[100,101] Compound **181** showed strong cytotoxic activity against several cancer cell lines.

One unusual pyran-ring containing cladiellane diterpene designated tritoniopsin B **182** was isolated from both the nudibranch *Tritoniopsis elegans* and its soft coral prey *Cladiella kremp*.[102] Bioassay-guided fractionation of extracts from a Fijian red alga in the genus *Callophycus* provided one new compound of the diterpene-benzoate class, bromophycoic acid C **183**, which exhibited modest activities against methicillin-resistant *Staphylococcus aureus* and the human malaria parasite *Plasmodium falciparum*.[103] Two xeniaphyllane peroxides gibberosins B and C **184** and **185** were isolated from a Taiwanese soft coral *Sinularia gibberosa*.[104] Six further members of this family containing the unusual cyclic peroxyhemiketal moiety, sinugibberosides A–F **186**–**191**, have been reported from the same species, *S. Gibberosa*.[105] It is conceivable that the biogenesis of these compounds derives from intramolecular cyclisation of a hydroperoxide structurally related to gibberosin B. The Formosan soft coral *Xenia umbellata* collected in Taiwan,

China, contained a cytotoxic xenicane diterpenoid xeniolide G **192**.[106] One meroditerpenoid, stypohydroperoxide **193**, was obtained from *Stypopodium flabelliforme* (Long Island, Papua New Guinea).[107]

One cytotoxic bromoditerpene **194** and the related antibacterial bromoditerpene 2*S*-hydroperoxy-12*R*-hydroxy-isobromosphaerol **195** were successively isolated from the

**161**

**162**

**163**

**164**

**165**

**166**

**167**

**168**

**169**

**170**

**171**

**172** $R_1$ = Ac
**173** $R_1$ = $COC_3H_7$

**174**

**175**

**176**

**177**

**178**

**179**

**180**

**181**

same collection of *Sphaerococcus coronopifolius* by the same group. The structure of the previously reported 12*S*-hydroxy-bromosphaerodiol[108] and 2*S*,12*S*-dihydroxyisobromo-sphaerol[109] were revised to **196** and **197**, respectively. The absolute stereochemistry of **194** was established by X-ray crystallographic analyses.[110,111]

182

185

191

183

186 R = OAc
187 R = OH
188 R = H

192

184

189 R = OAc
190 R = H

193

194

195

196

197

198

199

200

201

202

203

Chemical investigation on the gorgonian coral *Briareu*m sp. yielded a hydroperoxybriarane diterpene named briarenolide B **198** with a rare 9-ketobriarane moiety.[112] The same group afforded a further related briarenolide D **199** from a cultured specimen of the same organism.[113] Four diterpene compounds

**204**

**205**

**206**

**207** 11*R*
**208** 11*S*

**209** $R_1$ = H, $R_2$ = OOH
**210** $R_1$ = OOH, $R_2$ = H

**211** $R_1$ = OOH, $R_2$ = $CH_3$
**212** $R_1$ = $CH_3$, $R_2$ = OOH

**213**

**214**

**215**

**216**

**217**

**218**

**200–203** representing a new skeletal type, the dactylomelanes, have been found from specimens of *Laurencia* sp.[114]

A large number of highly functionalized cembranoid diterpenes and related metabolites have been isolated and identified from marine soft corals, especially from the genera *Lobophytum*, *Sarcophyton*, and *Sinularia.* A hydroperoxy-substituted cembranoid diterpene, 2-hydroperoxysarcophine **204**, was isolated from South-China-Sea soft coral *L. crassum*. It remains unclear whether **204** is a true natural product or an artifact.[115] One further cembranoid, crassumolide E **205**, was found from the same species.[116] A Kenting (Taiwan) collection of *Sinularia flexibilis* contained the cembranoid hydroperoxide flexilarin C **206**.[117] The same group provided two further structurally-related ε-lactones sinuladiterpenes A **207** and B **208** from the same species.[118] The Taiwanese soft coral *S. manaarensis* contained four cembrane-type diterpenoids, manaarenolides A **209** and B **210** and manaarenolides E **211** and F **212**, which were discovered for the first time as the hydroperoxycembranolides possessing a δ-lactone ring.[119] Four γ-cembranolide-type diterpenes, uprolides H–J **213–214**

**219**

**220**

**221**

**222**

and L **215** and M **216**, were reported from *Eunicea pinta* collected from San Andrés Island, Colombia. This study also led to the revision of the structures for nine previously reported uprolide B, uprolide B acetate, 8-*epi*-uprolide B, uprolide C acetate, 8-*epi*-uprolide B acetate,[120] 12,13-bis-epiuprolide B, 12,13-bisepiuprolide B acetate, uproeunicin, and uprolide C[121] to **217–225**, respectively.[122] Another compound of the type

**226** was isolated from the soft coral *Sarcophyton crassocaule* collected from the Xisha Islands in South China Sea. It exhibited strong cytotoxicity against the P388 cell line with an $IC_{50}$ value of 0.1 μg/mL.[123] The same source, *S. crassocaule*, provided three further cembranoid sarcocrassocolides F **227**, G **228**, and J **229**, all of which inhibited LPS-induced up-regulation of the pro-inammatory protein iNOS.[124] A chemical investigation of another species of the same genus, *S. Glaucum*, has led to the isolation of two peroxide diterpenes **230** and **231**, the absolute configuration of which were confirmed by X-ray diffraction and circular dichroism (CD) analyses. Compound **231** was found to be promising inhibitors of cytochrome $P_{450}$ 1A activity as well as inducers of GST and QR activity *in vitro* assays.[125]

A decalin-type bicyclic diterpenoid, lemnaloside C **232**, has been obtained from an extract of the marine soft coral *Lemnalia* sp.[126] The Japanese marine sponge *Epipolasis* sp. afforded a novel diterpene peroxypolasol **233**.[127] The Formosan soft coral *Nephthea pacifica* contained four prenylbicyclogermacrane diterpenoids, pacificins C **234**, E **235**, G **236**, and H **237**, of which **234** and **237** exhibited cytotoxicity against P388 cells with $ED_{50}$ of 1.44 and 2.01 μg/mL, respectively.[128]

**2.5 Other Marine Metabolites:** The Hainan Sponge *Dysidea septosa* contained a new sesquiterpene lingshuiperoxide **238**.[129] Three isothiocyanate sesquiterpenes axinisothiocyanates H **239** and I **240**, axinisothiocyanate N **241**, and aristolane derivative axinysone C **242** have been obtained from a sponge of the genus *Axinyssa* collected in the

**223**

**224**

**225**

Gulf of California by the same authors. Axinisothiocyanate N were mildly cytotoxic.[130,131]

Hydroperoxides have rarely been found in algae: two examples, dictyohydroperoxide **243** and hydroperoxy-acetoxycrenulide **244**, were isolated from *Dictyota dichotoma* (Troitsa Bay, Sea of Japan, Russia).[132] A aromandendrane sesquiterpenoid **245** was isolated from the Formosan soft coral *ClaVularia inflata*.[133] Chemical investigations of the soft coral *Nephthea erecta* have afforded three new sesquiterpenoids **246–248**, of which, **247** and **248** exhibited significant cytotoxicty against P388 and HT-29.[134] The Formosan soft coral *Nephthea erecta* provided the sesquiterpenoid **249**.[135] Five sesquiterpene peroxides sinularioperoxides **250–254** have been isolated from a Formosan soft coral of the genus *Sinularia* by the same group.[136,137]

An unusual 1,2-dioxolane-3-ol-containing sesquiterpene,

**226**

**227** $R_1$ = α-OOH, $R_2$ = OAc
**228** $R_1$ = β-OOH, $R_2$ = OAc
**229** $R_1$ = H, $R_2$ = OAc

**230**

**231**

**232**

**233**

**234**

**235**

**236**

**237**

**238**

**239** $R_1$ = *i*-Pr, $R_2$ = OOH
**240** $R_2$ = OOH, $R_1$ = *i*-Pr

**241**

**242**

**243**

**244**

**250**

**245**

**246** R = OH
**247** R = OOH

**248**

**249**

**251**

**252**

**253**

**254**

**255**

**256**

dioxosarcoguaiacol **255**, was reported from an Egyptian (Red Sea) collection of *Sarcophyton glaucum*.[138] A *Dysidea* sp. from the Great Barrier Reef contained a cytotoxic sesquiterpene **256**, the structure of which was determined by single crystal X-ray analysis.[139] Bioassay-guided fractionation of the Okinawan marine sponge *Dysidea chlorea* afforded two tricyclic spiro-sesquiterpenes, haterumadysins C and D **257** and **258**, both of which may be isolation artifacts.[140] One cuparene-derived sesquiterpene, laureperoxide **259**, has been reported from the red alga *Laurencia okamurai*.[141] The guaiane derivative peroxygibberol **260** has been obtained from the Formosan soft coral, *Sinularia gibberosa*, which was found to exhibit moderate cytotoxicity toward a human liver carcinoma cell line.[142]

The sipholane-type triterpenoids, sipholenol M **261**, siphonellinol E **262**, and siphonellinol hydroperoxide **263**, were isolated from the red sea sponge *Callyspongia* (*Siphonochalina*) *siphonella*.[143] Although there are several documented natural plant-derived triterpene hydroperoxides in the literature, it is also plausible that these three compounds are artifactual oxidation by products generated during the extraction and isolation process. *Bruguiera gymnorrhiza* yielded a dammarane-skeletoned triterpene bruguierin C **264** that activated antioxidant response element with micromolar potency.[144]

**257**

**258**

**259**

**260**

**261**

**262**

**263**

**264**

**265**

**266**

A Mediterranean collection of *Placida dendritica* afforded an unprecedented hydroperoxide **265**. Whether the hydroperoxide is an artifact of isolation, or a true natural product is unclear.[145] One halogenated nonterpenoid $C_{15}$-acetogenin, laurendecumenyne A **266**, has been reported from the Marine Red Alga *Laurencia decumbens*.[146]

Dihalenaquinolides A **267** and B **268**, from the Taiwanese marine sponge *Petrosia elastica*, have an unusual peroxide linkage between two meroterpenoid units.[147] Bioassay-guided fractionation of the marine cyanobacterium *Lyngbya* sp. led to the isolation of biselyngbyasides C **269** and D **270**, whose stereochemistries were established based on NOESY spectra and CD data.[148]

Two prenylated indole diketopiperazine alkaloids, spirotryprostatin E **271** and 13-oxoverruculogen **272**, have been obtained from the fermentation of *Aspergillus fumigatus* from a holothurian, *Stichopus japonicus* (Lingshan Is., Qingdao, China).[149] The antimalarial gracilioether A **273**, from the sponge *Agelas gracilis* (Oshima-Shinsone, Japan), are of mixed acetate/butanoate origin.[150] The sponge *Plakinastrella mamillaris* was a new source for gracilioether A **273**.[151] The same source, *P. Mamillaris*, afforded additional antimalarial gracilioether H **274** structurally related to gracilioether A. The existence of endoperoxide ring is important for the antimalarial activity.[152]

A collection of the sacoglossan *Placobranchus ocellatus* from the Philippines provided three propionate-derived metabolites, tridachiapyrone J **275**, and tridachiahydropyrones B **276** and C **277**, all of which are probably artifacts from oxidation during storage or workup.[153] Several years later, tridachiahydropyrones B and C were proved to be the same compound characterized as **278**.[154] The same species, *P. ocellatus*, provided the possibly artefactual peroxy derivative **279**,[155] whose relative configuration was confirmed at the same year.[156] A Panamanian collection of the sacoglossan mollusc *Elysia diomedea* yielded the endoperoxide **280**, structurally closely related to **279**.[157] The observation of rearrangement of **280** with triethylamine to yield the known vicinal diexpoxide elysiapyrone A[158] prompted speculation of the biosynthetic intermediate of **280**, likely to be in turn derived from a putative polypropionate alkenyl chain-containing precursor reacting with singlet oxygen.

## 3 Terrestrial Sources

**3.1 Monoterpenoids:** One *p*-menthane hydroperoxide, (1*R*,4*S*)-1-hydroperoxide-*p*-menth-2-en-8-ol-acetate **281** with strong trypanocidal avtivity, was isolated from the leaves of *Laurus nobilis*.[159] The same group afforded four further monoterpene hydroperoxides **282–285** with trypanocidal activity from *Chenopodium ambrosioides*. These hydroperox-

**267** R = $CH_3$
**268** R = $CH_2CH_3$

**269**

**270**

**271**

**272**

**273**

**274**

**275**

**276**

**277**

**278**

**279**

**280**

ides are likely formed through the singlet-oxygen oxidation of limonene, and the hydroperoxy group is essential for their trypanocidal activities.[160] The liverwort *Riella helicophylla* yielded six new monoterpenes **286–291**.[161] The aerial part of *Aster scaber* afforded two monoterpene peroxide glycosides **291–293**.[162] A cyclic monoterpene peroxide **294** with the irregular santolinyl framework was found from aerial parts of *Artemisia fragrans*.[163] The complete stereostructure of **295** has been established by application of the modified Mosher method.[164]

Catharoseumine **296**, a monoterpenoid indole alkaloid possessing a unique peroxy bridge moiety, was isolated from the whole plants of *Catharanthus roseus*. Its absolute configuration was determined by ECD and chemical methods. Catharoseumine exhibited cytotoxicity against HL-60 cell line with $IC_{50}$ value of 6.28 μM and potential inhibition against *Plasmodium falciparum* falcipain 2 ($IC_{50}$ = 4.06 μM). A plausible biogenetic pathway of catharoseumine was also proposed.[165]

### 3.2 Sesquiterpenes

**3.2.1 Guaianes:** Three highly oxygenated guaianolides **297–299** were isolated from the aerial parts of *Ajania*

281

282

283

284

285

286

287

288

289

290

291

294

292

293

295

296

*fruticulosa*. Compund **299** was inhibitory to the growth of *Candida albicans* with MICs being 20 µg/mL.[166,167] The aerial parts of *Achillea setacea* afforded a guaianolide **300** containing an endoperoxide ring.[168] Two guaianolides, anthemolide B **301** and 8-*O*-angeloyl-9-*O*-acetylanthemolide B **302**, were identified from the aerial parts of the flowering plant *Anthemis cretica*.[169] A cytotoxic sesquiterpene lactone, lactucin-8-*O*-p-methoxyphenyl acetate **303**, has been obtained from *Mulgedium tataricum*.[170] The structure of 1*α*,8*α*-epidioxy-4*α*-hydroxy-5*α*H-guai-7(11),9-dien-12,8-olide **304**, isolated from *Curcuma wenyujin* with anti-influenza viral activity, has been confirmed by single-crystal X-ray diffraction experiment.[171] The complete relative configuration of the known sesquiterpene (+)-dioxo-sarcoguaiacol has been established. This compound has now been isolated from *Acorus calamus*.[172]

Chemical examinations of the roots of *Nardostachys chinensis* afforded two antimalarial guaiane endoperoxides, nardoperoxide **305** and isonardoperoxide **306**, whose absolute stereochemistries were determined by CD spectra. The endoperoxide moiety of the molecules was assumed to relate to the antimalarial activity.[173] A subsequent report described another four related endoperoxides nardoguaianones A–D **307–310** from the same plant.[174] Three hydroperoxides **311–313** with trypanocidal activity have been isolated from *Pogostemon cablin*,[175] whilst the sesquiterpene peroxide **314** has been found from the aerial parts of *Croton arboreous*.[176]

**3.2.2 Eudesmanes:** The aerial parts of *Montanoa hibiscifolia* afforded three eudesmanolides **315–317** with a rare endoperoxide structural element.[177] The novel eudesmanolide **318** has been isolated from *Atractylodes macrocephala*.[178] The aerial parts of *Aster spathulifolius* was the source for two cytotoxic sesquiterpene hydroperoxides, 7*α*-hydroperoxy-3,11-eudesmadiene **319** and 7*β*-hydroperoxy-eudesma-11-en-4-ol **320**.[179,180] The sesquiterpene schisansphene A **321** was identified from the species *Schisandra sphenanthera*.[181] A eudesmane derivative hydroperoxy-gynuradiene **322** has been obtained from the root of *Gynura bicolor*.[182] Another two compounds of this type **323** and **324** were discovered from *Xylopia emarginata* and *Ecdysanthera rosea*, respectively.[183,184]

Two novel eudesmene-type sesquiterpene peroxides, kandenols C **325** and D **326**, have been reported from *Streptomyces* sp. derived from the mangrove plant *Kandelia candel*.[185] The aerial parts of *Inula japonica* contained two eudesmane sesquiterpenoids **327** and **328**. Compound **328** was confirmed by means of single-crystal X-ray diffraction analysis.[186] One eudesmane derivative **329** has been isolated from the liverworts *Chiloscyphus polyanthus*.[187] Other eudesmane peroxides included 1*β*,14-peroxy-4*α*-hydroxy-5*α*H,7*α*H,6*β*H-eudesm-11(13)-en-6,12-olide **330** from the roots of *Vladimiria souliei*,[188] 3*α*-dehydroxy-3*α*-hydroperoxy-clypeotriol **331** from *Achillea clypeolata*,[189] and 5*α*-hydroperoxy-eudesma-4(15),11-diene **332** from *Artemisia annua*.[190]

**3.2.3 Bisabolanes and Germacranes:** Four bisabolane-type sesquiterpenes, peroxylippidulcines A–C **333–335** and

**297**

**298**

**299**

**300**

**301** $R_1 = H, R_2 = H$
**302** $R_2 = Ang, R_2 = Ac$

**303**

**304**

**305**

**306**

**307**

**308**

**309**

**310**

**311**

**312**

**313**

**314**

peroxyepilippidulcine B **336**, have been obtained from the aerial parts of *Lippia dulcis*. The relative configurations of **334** and **336** were confirmed by X-ray crystallographic analysis data.[191] The aerial parts of *Carthamus lanatus* afforded two oxygenated bisabolane fucosides **337** and **338**.[192] Another species of the genus, *C. glaucus*, contained two bisabolane fucopyranosides **339** and **340**.[193] Another bisabolene derivative **341** was found from the aerial parts of *Achillea clavennae*.[194]

A germacranolide peroxide **342** was identified as a component of *mulgedium tataricum*.[170] Chemical investigation of *Santolina insularis* afforded two germacrane sesquiterpene peroxides **343** and **344**, which might derive from the formal photo-oxygenation of the corresponding $\Delta^{4,5}$ olefin, a reaction well precedented in medium-sized olefins.[195]

**3.2.4 Sesquiterpene Dimers:** A dimeric sesquiterpene lactone japonicone E **345** bearing a rare hydroperoxide group was obtained from the aerial parts of *Inula japonica*, which displayed strong inhibitory activity against LPS-induced NO production in RAW264.7 macrophages.[196] Further investigations of the same species afforded additional related dimeric sesquiterpene, japonicone T **346**.[197] The leaves of *Xylopia vielana* contained a dimeric guaiane peroxide named vielanin C **347** with a central cyclobutane ring that are generated from two equal guaiane moieties by [2 + 2] cycloaddition.[198] Two further related vielanins D **348** and E **349** were isolated from the same plant as epimeric mixtures. Both compounds consist of bridged ring systems formally representing the Diels-Alder products from the hypothetical guaiane-type monomers.[199] Spicachlorantins C–F **350–353**, new lindenane sesquiterpene dimers possessing a hydroperoxy group, were isolated from the roots of *Chloranthus spicatus*, whose absolute stereostructures were established by CD spectroscopic analyses. These compounds were considered to be biogenetic precursors of the corresponding hydroxyl derivatives of dimeric lindenane sesquiterpenoids distributed in *Chloranthus* plants.[200] Another species of the genus, *C. Japonicus*, contained one more dimeric sesquiterpene peroxide **354**, structurally related to **350–353**.[201]

**315**

**316**

**317**

**318**

**319**

**320**

**321**

**322**

**323**

**324**

**325**

**326**

**327**

**328**

**329**

**330**

**331**

**332**

**333**

**334**

**335**

**336**

**337**

**338**

**339** R =
**340** R =

**341**

**342**

**343** R = H
**344** R = Ac

**3.2.5 Other Sesquiterpenes:** The structures of cytosporolides A–C **355–357**[202] have been revised on the basis of synthetic studies and reinterpretation of the NMR data. Cytosporolide A, which was originally assigned the strained nine-membered peroxylactone structure, has been revised to **358**, which is probably biogenetically formed by a hetero-Diels-Alder type cyclization.[203]

**345**

**346**

**347**

**348**

**349**

**350** $R_1$ = OOH, $R_2$ = Me, $R_3$ = H
**351** $R_1$ = OOH, $R_2$ = H, $R_3$ = Me

**352** $R_1$ = OH, $R_2$ = Ac, $R_3$ = Tig
**353** $R_1$ = H, $R_2$ = H, $R_3$ = Ac

**354**

**355** $R_1$ = OH, $R_2$ = $OCH_3$, $R_3$ = H
**356** $R_1$ = H, $R_2$ = OH, $R_3$ = H
**357** $R_1$ = OH, $R_2$ = $OCH_3$, $R_3$ = Ac

**358**

The novel norsesquiterpene peroxides steperoxides A–D **359–362** have been obtained from the mushroom *Steccherinum ochraceum*,[204,205] while another nor-chamigrane merulin A , and the chamigranes merulins B–D **363–365**, have been found in an extract of the culture broth of a Thai mangrove-derived fungus.[206,207] We have observed that steperoxide B and merulin A have the same structure **360**. Among these isolated metabolites, merulin C exhibited potent antiangiogenic activity. Another four compounds of this type, talaperoxides A–D **366–369**, have been obtained from *Talaromyces flavus*. Talaperoxides B and D were moderately cytotoxic to several human tumour cell lines.[208] The structures of **359**, **360**, **366** and **367** were further confirmed by X-ray crystallographic analysis, and the absolute configurations of the latter three compounds were also determined using copper radiation[204,206,208]

Five peroxy cuparene-type sesquiterpenoids **370–374** were identified from the Japanese liverwort *Jungermannia infusca*. The stereostructure of **370** was confirmed by X-ray crystallographic analysis.[209,210] An inseparable diastereomeric mixture acetylmajapolene A **375** in the part of the peroxide with antibacterial activity have been found in an extract of an undescribed Malaysian species of the *Laurencia* genus, whose absolute configurations have been unambiguously determined as (1*R*,4*R*,7*S*,10*S*) and (1*S*,4*S*,7*S*,10*S*), respectively, by vibrational circular dichroism (VCD).[211,212]

Two novel muurolane sesquiterpene peroxides, 1,4-peroxy-muurol-5-ene **376** and 1,4-peroxy-5-hydroxy-muurol-6-ene **377** have been obtained from *Illicium tsangii*. The absolute stereochemistry of **376** was confirmed by X-ray crystallography.[213] A peroxy muurolane-type sesquiterpenoid **378** was isolated from the Belgium liverwort *Scapania undulata*.[214] The essential oil of the liverwort *Plagiochila asplenioides* contained one oxygenated sesquiterpene (+)-muurolan-4,7-peroxide **379**.[215] The NMR data of the sesquiterpene peroxide **380**[216] are also reported for the first time in the same paper. The aerial parts of the invasive plant *Eupatorium adenophorum* contain the new sesquiterpene **381**.[217] Dihydroartemisinic acid hydroperoxide **382** was isolated for the first time as a natural product from the plant *Artemisia annua*. The compound is a probable precursor of artemisinin under nonenzymatic conditions.[218] The same plant, *A. annua*, afforded a rare seven-membered endoperoxide lactone arteannuin H **383**, a biomimetic synthesis of which has confirmed biogenetic speculations regarding its formation from a secondary allylic hydroperoxide.[219,220] The structure of **384**, isolated from the leaves of *Eupatorium adenophorum*, was determined by single-crystal X-ray crystallography.[221]

A phytochemical study of *Robinsonecio gerberifolius* afforded a eremophilane derivative **385**, whose absolute configuration was established from CD analysis.[222] Three species of the *Ligularia* genus, *L. subspicata*, *L. Kanaitzensis*, and *L. Veitchiana*, provided the eremophilane peroxides **386**, **387**, and **388**, respectively.[223–225] Another compound of this type **389** was isolated from *Cacalia tangutica*.[226]

The aerial parts of *Anthemis arvensis* contained two irregular linear sesquiterpene lactones **390** and **391**, both of which were re-isolated from the same plant by another group of researchers.[227,228] A different species of *Anthemis*, *A. cotula*, afforded additional related peroxide, 5-hydroperoxy-6,13-dehydro-5,6-dihydroanthecotuloide **392**.[229]

Three isomeric sesquiterpene hydroperoxides **393–395** were isolated from *Illicium tsangii*. These compounds appear to be derived from the ene-type addition of molecular oxygen to the known compound *α*-santalene.[230] A bioassay-guided fractionation of extract from *Scleria striatinux* led to the

**359**

**360**

**361**

**362**

**363**

**364**

**365**

**366** R = αCH$_3$
**367** R = βCH$_3$

**368** R = αCH$_3$
**369** R = βCH$_3$

**370**

**371**

**372**

**373**

**374**

**375**

isolation of okundoperoxide **396**, a compound with antiplasmodial activity.[231]

The aerial parts of *Xanthium strumarium* contained one xanthane-type sesquiterpenoid, 4*β*,5*β*-epoxyxanthatin-1*α*,4*α*-endoperoxide **397**.[232] One allohimachalane peroxide **398** has been obtained from *Illicium tsangii.*[233] The extract of the aerial parts of *Artemisia diffusa* contains tehranolide **399**, a new type of sesquiterpene lactones with an endoperoxide group.[234] Successful biomimetic syntheses of the litseaverticillol family of sesquiterpenes have been achieved, using singlet oxygen chemistry.[235] In this work, the structure of the previously reported litseaverticillol E[236] has been revised to **400**.

Artemisinin, the well-known antimalarial agent, has been the focus of continuing study. Its antimalarial activity, structural modification, structure-activity relationships, mode of actions, and use in therapy have been well reviewed.[237–240]

**3.3 Diterpenes:** A dolabellane diterpene derivative **401** with the naturally rare peroxy function was identified as a component of the aerial parts of *Cleome droserifolia*,[241] and additional related peroxide **402** was found from *Aglaia odorata*.[242] *Jatropha integerrima* provided a rhamnofolane endoperoxide 2-epicaniojane **403**, whose structure was confirmed by X-ray diffraction analysis.[243]

A clerodane peroxide, 15(16)-peroxy-3,13-clerodadien-18-oic acid **404**, was isolated from the Taiwanese liverwort *Schistochila acuminata*,[244] and the structurally related 2*β*-hydroperoxykolavelool **405** was reported from *Aristolochia chamissonis*.[245] The plant *Casearia arguta* afforded further members of the series, argutins F–H **406–408**.[246]

The aerial parts of *Aster oharai* contained two labdane peroxides **409** and **410**, of which compound **409** showed moderate cytotoxicity against several human tumor cell lines with $ED_{50}$ values ranging from 1.1 to 7.7 μg/mL.[247] A different species of *Aster*, *A. spathulifofius*, provided further related 7*α*-hydroperoxymanool **411** that showed moderate cytotoxicity against human cancer cells.[179] Other compounds of this type included (8*S*)-hydroperoxy-(13*S*)-hydroxy-9(11),14-labdadiene **412** from *Jungermannia infuscua*,[210] *ent*-12,15-dioxo-3,4-*seco*-4,8,13-labdatrien-3-oic acid **413** and *ent*-12,15-dioxo-8,13-labdadien-3*α*-ol **414** from *Croton stipuliformis*,[248] and 8*α*-hydroxy-13-hydroperoxylabd-14,17-dien-19,16:23,6*α*-diolide **415** from *Salvia sahendica*.[249] The absolute stereochemistry of compound **414** was determined by application of Mosher's method.

**376**

**377**

**378**

**379**

**380**

**381**

**382**

**383**

**384**

**385**

**386**

**387**

**388**

**389**

**390**

**391**

**392**

**393**

**394**

**395**

**396**

**397**

**398**

**399**

**400**

The leaves of *Viburnum awabuki* afforded two vibsane hydroperoxides vibsanin K **416** and 18-*O*-methylvibsanin K **417** as well as their corresponding C-5 epimers **418** and **419**,[250,251] of which vibsanin K exhibited significant cytotoxicity against human gastric (NUGC) and oral epidermoid (HONE-1) tumor cells at a concentration of 50 μg/mL.[252] An unusual macrocyclic endoperoxide structure was assigned to neovibsanin C **420** that was obtained from *Viburnum aurabuki*.[253] Two cytotoxic diterpenes, dysokusones B **421** and C **422**, were isolated from the stem of *Dysoxylum kuskusense*.[254] A rare open chain peroxide designated

**401**

**402**

**403**

**404**

**405**

**406** $R_1$ = X, $R_2$ = OH, $R_3$ = H
**407** $R_1$ = X, $R_2$ = OH, $R_3$ = OH
**408** $R_1$ = OH, $R_2$ =X, $R_3$ = OH

**409**

**410**

**411**

**412**

**413**

**414**

**415**

**416**

**417**

**418** $R_1$ = OOH, $R_2$ = H
**419** $R_1$ = OOH, $R_2$ = $CH_3$

**420**

**421**

**422**

**423**

leucoperoxyterpene **423** with good antibacterial activity has been isolated from aerial parts of the medicinal plant *Leucosceptrum canum*.[255]

Jungermatrobrunin A **424**, which was obtained from the liverwort *Jungermannia atrobrunnea*, has an unusual rearrangedent-kaurene skeleton with a peroxide bridge. Its relative configuration was further supported by a single-crystal X-ray crystallographic analysis.[256] A phytochemical investigation on the stems of *Annona squamosa* led to the isolation of additional two *ent*-Kaurane hydroperoxides, annosquamosins F **425** and G **426**.[257]

The leaves of *Croton steenkampianus* provided a novel diterpenoid steenkrotin B **427**, which possess a new carbon skeleton that may be derived from the daphnanetype by an 8(9→10)-*abeo* rearrangement.[258] A rare 3,4-*seco*-cleistanthane hydroperoxide designated as trigonochinene C **428** with anti-microbial activity was isolated from the aerial parts of *Trigonostemon chinensis*.[259]

Nine jatrophane hydroperoxides, amygdaloidins C **429** and E–L **430**–**437**, have been isolated from the wood spurge, *Euphorbia amygdaloides*.[260] A methanol extract of *Anisomeles indica* afforded two cembrane hydroperoxides 4-methylene-5$\beta$-hydroperoxyovatodiolide **438** and 4$\alpha$-Hydroperoxy-5-enovatodiolide **439**, of which **439** showed inhibitory effects on antiplatelet aggregation induced by thrombin.[261]

Two abietane endoperoxides **440** and **441** were isolated as the corresponding acetate derivatives from the cones of *Cedrus atlantica*.[262] The aerial parts of *Illicium angustisepalum* contained four more abietane diterpenes, angustanoic acids B–D **442**–**444** and I **445**.[263] Investigation of the leaves and twigs of *Callicarpa longissima* resulted in the isolation of a 3,4-*seco*-abietane peroxide named callilongisins A **446** with significant anti-inflammatory effect, whose structure was further confirmed by X-ray crystallographic analysis.[264] Three diterpenic acids **447**–**449** were isolated as their methyl ester derivatives from the leaves of *Juniperus thurifera* and

424

425 R = $CH_2OCOCH_3$
426 R = $CH_2OH$

427

428

Nic =

Ang =

Hydrp =

**429** $R_1$ = Hydrp, $R_2$ = H, $R_3$ = Ang, $R_4$= Ac, $R_5$ = Nic
**430** $R_1$ = Ang, $R_2$ = H, $R_3$ = Hydrp, $R_4$= Ac, $R_5$ = Ac
**431** $R_1$ = Ang, $R_2$ = Ac, $R_3$ = H, $R_4$ = Hydrp, $R_5$ = Ac
**432** $R_1$ = Hydrp, $R_2$ = Ac, $R_3$ = H, $R_4$ = Ang, $R_5$ = Ac
**433** $R_1$ = Ac, $R_2$ = Hydrp, $R_3$ = H, $R_4$ = Ang, $R_5$ = Ac
**434** $R_1$ = Hydrp, $R_2$ = Ac, $R_3$ = Ang, $R_4$ = H, $R_5$ = Ac
**435** $R_1$ = Ac, $R_2$ = Hydrp, $R_3$ = Ang, $R_4$ = H, $R_5$ = Ac
**436** $R_1$ = Ang, $R_2$ = Ac, $R_3$ = Hydrp, $R_4$ = H, $R_5$ = Ac

437

*Juniperus phoenicea*.[265] Further members of the type included triptotins A **450** and B **451** from *Tripterygium wilfordii*,[266] 6-oxo-12-peroxyabieta-8,11,13-triene **452** from *Salvia multicaulis*,[267] and glutinosin C **453** from *Isodon glutinosa*.[268] The structures of triptotin A and glutinosin C were confirmed by single crystal X-ray analysis. Phytochemical investigation of the above-ground parts of *Siegesbeckia pubescens* yielded one *ent*-pimarane diterpenoid **454**.[269]

**3.4 Triterpenes:** A taraxastane-type triterpene, 3*β*-acetoxy-19*α*-hydroperoxy-20-taraxastene **455**, has been isolated from the aerial roots of *Ficus microcarpa*.[270] Reinvestigation of the aerial root extract afforded five ursene derivatives **456**–**460**.[271,272] The structure of **460** was confirmed by X-ray crystallography. Another compound of this type **461** were obtained from *Arnica montana*.[273] The rhizome of *Vladimiria muliensis* provided one antimicrobial ursane triterpenoid 1*α*,5*α*-dioxy-11*α*-hydroxyurs-12-en-3-one **462**.[274] Other ursene triterpenoids were including 3*β*,28-dihydroxy-11*α*-hydroperoxy-12-ursene **463** from *Tolpis proustii*,[275] speciosaperoxide **464** from *Chaenomeles speciosa,*[276] and (2*β*,3*β*)-3,25-epidioxy-2,24-dihydroxyursa-12,20(30)-dien-28-oic acid **465** and (2*β*,3*β*)-3,25-epidioxy-2,24-dihydroxyurs-12-en-28-oic acid **466** from *Gentiana aristata.*[277]

Ginsenoside $SG_2$ **467** has been reported from black ginseng.[278] A pair of allylic hydroperoxides, ginsneoside-$Rh_6$ **468** and floralginsenoside ka **469**, were obtained from the leaves and flower buds of *Panax ginseng*, respectively. Floralginsenoside ka displayed potent scavenging activity with the inhibition value of 64% at 10 μM.[279,280] The same species contained six dammarane-type triterpene diglycosides, floralginsenosides A–F **470**–**475**, five dammarane triterpene triglycosides, floralginsenosides G–K **476**–**480**, and a dammarane triterpene obligoglycoside, ginsenoside SF **481**.[281–283]

Six dammarane triterpenes, named probosciderols D–I **482**–**487**, have been found in *Proboscidea louisiana*.[284] The stem bark of *Rhus javanica* contained a dammarane triterpene designated as isofouquierone peroxide **488**.[285] Ginsenosides I and II from *Panax ginseng* have new genins **489** and **490**.[286] The fruits of *Ceriops tagal* was the source for a dammarane triterpene cereotagaloperoxide **491**.[287] Aglaiabbreviatin F **492** was identified as a component of the stems of *Aglaia abbreviata*.[288] Another two compounds of this type **493** and **494** were isolated from the fruits of *Ligustrum lucidum.*[289]

One lanostane peroxide 5*α*,8*α*-peroxydehydrotumulosic acid **495** was isolated from the epidermis of the sclerotia of *Poria coco.*[290] Additional two compounds of this type, inoterpenes C **496** and E **497**, were discovered from the sclerotia of *Inonotus obliquus*.[291] The leaves of *Melaleuca ericifolia* was the source for two antiproliferative norlupane triterpenes **498** and **499**.[292] The aerial roots of *Ficus microcarpa* afforded another norlupane triterpene **500**.[272]

One novel 29-*nor*-3,4-*seco*-cycloartane triterpene methyl ester **501** was isolated from the aerial parts of *Antirhea acutata*, which showed moderate inhibitory activities in cyclooxygenase-1 and -2 assays.[293] Phytochemical investigation of the leaves of *Markhamia lutea* resulted in the isolation of two cycloartane triterpenoids, musambins A **502** and B **503**, as well as corresponding xylosides, musambiosides A **504** and B **505**. These compounds showed anti-plasmodial and anti-trypanosomal activity.[294] *Combretum quadrangulare* contained a novel cycloartane-type triterpene named methyl quadrangularate B **506** that exhibited potent cytotoxicity with

**438**

**439**

**440**

**441**

**442**

**443**

**444**

**445**

**446**

**447** R = OOH
**448** R =OH

**449**

**450**

**451**

**452**

**453**

**454**

a $ED_{50}$ value of 9.54 μM.[295] The same source afforded additional related quadrangularic acid F **507** by the same group.[296]

The aerial roots of *Ficus microcarpa* afforded two oleanane triterpenoids **508** and **509**. The structures of **508** was further confirmed by X-ray crystallography.[271,272] Another compound of this class, sarmentolin **510**, was identified as a hepatoprotective agent from *Sedum sarmentosum*.[297] A glutinane triterpene **511** was identified as a component of the aerial parts of *Maytenus apurimacensis*.[298] Aceranol acetate **512** was a 5,6-cleaved glutinane derivative from *Acer mandshuricum*.[299]

A peroxy-multiflorane triterpene ester **513** has been isolated from the processed seeds of *Trichosanthes kirilowii*.[300] The plant *Azadirachta indica* contained a tetranortriterpenoid, 4*α*-hydroperoxy-6-*O*-acetylnimbandiol **514**.[301] The absolute configuration of known longilene peroxide[302], isolated from the wood of *Eurycoma longifolia*, has been established by total synthesis.[303]

Two euphane hydroperoxides, meliasenins A **515** and C **516**, were isolated from the stem bark of *Melia toosendan*.[304] Further members of this type, meliasenins I–O **517**–**523**, were obtained from the fruits of the same plant. The relative configuration of **517** was further confirmed by single-crystal X-ray diffraction analysis.[305] Another two species of this genus, *M. dubia* and *M. azedarach*, contained meliastatin **524** and 25-hydroperoxytirucalla-7,23(24)-diene-3,6-dion-21,16-olide **525**, respectively. Meliastatin exhibited significant inhibition of the P388 cancer cell line.[306,307] The roots of *Euphorbia micractina*

**455**

**456** $R_1$ = Ac
**457** $R_1$ = H

**458**

**459**

**460**

**461**

**462**

**463**

**464**

**465**

**466**

afforded further euphane/tirucallane derivatives **526–530**.[308]

Three 3(4),9(10)-disecocycloartane peroxy triterpene lactones, pseudolarolides $Q_2$ **531**, $T_1$ **532**, and $T_2$ **533**, were discovered from the seeds of *Pseudolarix kaempferi*.[309] The leaves of the same species contained three more triterpene peroxides, pseudolarolides Q–S **534–536**. The stereochemical structures of these compounds were confirmed by single-crystal X-ray analyses.[310] One triterpene dilactones with a rare rearranged pentacyclic skeleton, longipedlactone K **537**, was found from the stems of *Kadsura ananosma*.[311] A cytotoxic triterpenoid schinalactone A **538**, an endoperoxide with an unusual contracted ring A, has been isolated from the roots and stems of *Schisandra sphenanthera*, which showed significant cytotoxicity against PANC-1 cell lines with a $IC_{50}$ value of 5.9 μM.[312] The structure of a non-peroxidic metabolite, named podocarpaside E,[313] has been revised to **539** on the basis of an X-ray analysis.[314]

**3.5 Others:** The structurally novel antiproliferative metabolite designated hexacyclinol **540** was first described by Gräfe and co-workers from basidiospores collected from *Panus rudis* growing on dead betula woods in Siberia.[315] The structure of hexacyclinol was subsequently revised, and an alternative structure **541** was confirmed *via* total synthesis. In addition, an X-ray crystal structure was obtained, providing unequivocal structural confirmation.[316,317] The first peroxide among the prenylated benzophenones, plukenetione C **542**, was reported from the fruits of *Clusia plukenetii*.[318] Continuing

**468**

**467** $R_1$ = Glc, $R_2$ = H
**469** $R_1$ = H, $R_2$ = Glc

**470**

**471**

investigations of the plant yielded two further related prenylated benzophenone derivatives, 33-hydroperoxyiso-plukenetione C **543** and 15,16-dihydro-16-hydroperoxy-plukenetione F **544**.[319] Another two compounds of this type, peroxysampsones A **545** and B **546**, were isolated from the roots of the Chinese medicinal plant *Hypericum sampsonii*, of which peroxysampsone A showed comparable activity with norfloxacin against a NorA over-expressing multidrug-resistant (MDR) strain of *Staphylococcus aureus* SA-1199B.[320]

**472**

**473**

**474**

**475**

**476**

**477**

**478**

**479**

**480**

**481**

A neurofibromatosis type 1 (*NF1*)-based bioassay-guided phytochemical investigation on *Zanthoxylum armatum* collected in Nepal led to the isolation of two isomeric timuramides A **547** and B **548**, both of which can inhibite growth of *Nf*1-defective tumor cell line at noncytotoxic concentrations.[321] One antibacterial acylphloroglucinol, olympicin D **549**, was isolated and characterized from the aerial parts of *Hypericum olympicum*.[322] A hydroperoxyquinolone alkaloid, glycopentaphyllone **550**, was reported from the fruits of *Glycosmis pentaphylla*, whose absolute configuration was established by applying Mosher's method.[323]

Walsuronoid A **551** was the first limonoid with a peroxide linkage from *Walsura robusta.* The structure of walsuronoid A was also confirmed by X-ray analysis.[324] The stems of *Khaya anthotheca* afforded one further limonoid **552**,[325] and the related xylocarpin G **553** was obtained from the Chinese mangrove plant, *Xylocarpus granatum*.[326] Additional member of the group, munronoid F **554**, was discovered from *Munronia unifoliolata*.[327]

Two unprecedented spiroketal peroxides, chloropupukeanolides A **555** and B **556**, were isolated from an endophytic fungus *Pestalotiopsis fici*, with chloropupukeanolide A showing significant anti-HIV-1 and cytotoxic effects. A possible biosynthetic pathway to chloropupukeanolides A and B has been proposed.[328] A cytotoxic prenylated flavone, named artoindonesianin B **557**, was obtained from the root of *Artocarpus champeden*.[329] The root of *Zanthoxylum zanthoxyloides* provided an aromatic peroxide **558**.[330]

A peroxy acid urticic acid **559** was discovered from the whole plant of *Leucas urticifolia*.[331] A spiranoid withanolide **560** was obtained from the leaves of *Jaborosa odonelliana*.[332]

**482**

**483**

**484**

**485**

**486**

**487**

**488**

**489** R = α-OOH
**490** R = β-OOH

**491**

**492**

**493** R = Ac
**494** R = H

**495**

**496**

**497**

**498** R = βOOH
**499** R = αOOH

The stems of *Millettia taiwaniana* contained one isoflavonoid peroxide millewanin E **561**.[333] Brasixanthone C **562** was identified as a constituent of the stem bark of Calophyllum brasilienses collected in Brazil.[334] One lignan tiegusanin M **563** was a constituent of the aerial parts of *Schisandra propinqua*.[335] The unique neolignan mansoxetane **564**, isolated from the heartwood of *Mansonia gagei*, is the first example of a biphenylneolignan with a dioxetane ring discovered in nature.[336]

Two prenylated polyketides, harrisotones C **565** and D **566** representing a rare spirocyclic skeleton, along with a cytotoxic hydroperoxypolyketide harrisonol A **567**, were isolated from *Harrisonia perforata*.[337] Two butanolides, litseadioxanins A **568** and B **569** bearing a 1,2-dioxane moiety, were obtained from the stem bark of *Litsea akoensis*.[338]

Chemical investigation of the leaves of *Machilus japonica* resulted in the isolation of apigenosylides A–C **570**–**572**, which possess an unprecedented skeleton comprising the

500

501

502

503

504

505

506

507

508

509

510

511

512

513

514

adduct of a butenolide moiety and apigenin glycoside linked *via* a 1,2-dioxane moiety. Apigenosylides B–C possess moderate inhibitory activity against *α*-glucosidase.[339] High-throughput natural products chemistry methods have facilitated the isolation of a beilschmiedic acid peroxide beilschmiedic acid N **573** from the leaves of a Gabonese species of *Beilschmiedia*, which may be an artifact of isolation formed through Diels-Alder addition of singlet oxygen.[340] A cyclic peroxide named kramecyne **574** with good anti-inflammatory activity has been isolated from *Krameria cytisoides*.[341]

Xanthoangelol E, originally obtained from the root of *Angelica keiskei*,[342] showed the effects of xanthoangelol, on NF-κB activation and ET-1 gene expression in cultured porcine aortic endothelial cells.[343] Two furanocoumarins, melicotriphyllins B **575** and D **576** bearing a hydroperoxy group on the geranyloxy side chain, were isolated from the

**515**

**516**

**517** $R_1$ = H, $R_2$ = OH, $R_3$ = $CH_3$
**518** $R_1$ = O, $R_2$ = O, $R_3$ = $CH_3$

**519** $R_1$ = H, $R_2$ = OH, $R_3$ = OOH
**520** $R_1$ = O, $R_2$ = O, $R_3$ = OOH

**521**

**522** 24*S*
**523** 24*R*

**524**

**525**

**526** $R_1$ = β-Me, $R_2$ = β-OOH
**527** $R_1$ = β-Me, $R_2$ = α-OOH
**528** $R_1$ = α-Me, $R_2$ = α-OOH

**529**

**530**

fruits of *Melicope triphylla*.[344]

Two rare four-membered peroxide-containing pheophytin, bidenphytins A **577** and B **578**, were identified from *Biden pilosa,* a popular Taiwanese folk medicine. Possible biosynthetic pathways for them has been proposed.[345] Bioassay-guided fractionation of the extract from *Kielmeyera coriacea* afforded a novel *δ*-tocotrienol peroxy-dimer **579**.[346] Two dimeric anthrone peroxides, adxanthromycins A **580** and B **581**, were new inhibitors of ICAM-1/LFA-1 mediated cell adhesion molecule isolated from the fermentation broth of an

**531**

**532**

**533**

**534**

**535**

**536**

**537**

**538**

**539**

**540**

**541**

**542**

**543**

**544**

**545**

**546**

undescribed *Streptomyces* species.[347] The aerial parts of the medicinal plant *Clerodendrum bungei* afforded additional peroxide dimer named bungein A **582**.[348]

Clausamine G **583** containing a hydroperoxy moiety in the molecule, is the first example of the isolation of a peroxygen-

**547**

**548**

**549**

**550**

**551**

**552**

**553**

**554**

**555** R = $CH_3$
**556** R = H

**557**

**558**

ated carbazole alkaloid in nature.[349] The leaves of *Piper aduncum* afforded an prenylated benzoic acid derivative **584** with antifungal activity whilst the related **585** was obtained from the aerial parts of *Aster spathulifolius*. The presence of a hydroperoxide group at the side chain could be directly associated to its fungitoxicity.[179,350] Bioactivity-guided fractionation of the extract from *Piper crassinervium* afforded one prenylated hydroquinone **586**.[351]

The buds of *Lonicera japonica* contained a novel cyclic peroxide named shuangkangsu **587** with significant antiviral activities, whose absolute stereochemistry was determined by CD analysis.[352] Echinobithiophene A **588**, a peroxide bithiophene with significant antimicrobial activity, was isolated from *Echinops ritro*, and its structure was identified by spectral analysis including 2D NMR, and comparison of optical rotation values and chemical shifts of $^{13}$C NMR between the predicted and experimental data.[353] A pyrrolidone peroxide cucubalactam **589** has been reported from *Cucubalus baccifer*.[354] A mutualist actinomycete of the southern pine beetle, *Dendroctonus frontalis*, produced a polyene peroxide, mycangimycin **590**, with pronounced antifungal activity. Its absolute configuration was determined by chemical modification followed by the modified Mosher method.[355] The stem bark of the African tree *Antiaris africana* afforded a cardiac glycoside africanoside **591**, which effected a concentration-dependent inhibition of tumor cell growth with a mean $IC_{50}$ value of 5.3 nM.[356]

## 4 Steroidal Peroxides

The ubiquitous ergosterol peroxide[357] continued to be isolated from any number of sources, marine as well as terrestrial, particularly mushrooms. The diverse biological activities have been attributed to ergosterol peroxide. Ergosterol peroxide was found to be a inhibitor to the proliferation of K562, Jurkat, WM-1341, HL-60, and RPM1-8226 tumor cell lines by 10 to 40% at 10 μg/mL.[358] Ergosterol peroxide from

**559**

**560**

**561**

**562**

**563**

**564**

**565**

**566**

**567**

**568**

**569**

the marine sponge *Spirastrella abata* showed cytotoxicity against several human solid tumor cell lines,[359] and also against human gastric tumor cell line (SNU-1), human hepatoma cell line (SNU-354), human colorectal tumor cell line (SNU-C4), and murine sarcoma-180 were 18.7, 158.2, 84.6 and 74.1 μM ($IC_{50}$), respectively.[360] Ergosterol peroxide from two species of the *Pleurotus* genus, *P. eryngii* and *P. ostreatus*, exhibited osteoclastogenesis inhibitory and trypanocidal activity, respectively.[361,362] Ergosterol peroxide was obtained for the first time from *Oryza sativa* in 2006. This is the first report of potential allelopathic activity of steroids on weeds based on their phytotoxicity on barnyardgrass (Echinochloa crus-galli) as target species.[363] Ergosterol peroxide was found to be a DNA topoisomerase I inhibitor,[364] and exhibit potent of rat lens aldose reductase inhibition.[365] Among the lipophilic extracts of seven traditional edible mushrooms, the acetone extract of *Sarcodon aspratus* markedly inhibited the growth of HL60 human leukemia cells and induced apoptosis after 24 h incubation. The major active component was identified as ergosterol peroxide. It is completely inhibited growth and induced apoptosis of HL60 cells at a concentration of 25 μM.[366] Anti-inflammatory activity has been found for ergosterol peroxide isolated from several species.[367–369] Ergosterol peroxide also displayed strong anticomplement activity on the classical pathway with $IC_{50}$ values of 126.8 μM.[370] In addition, the antimicrobial,[371,372] antituberculosis,[373] and melanogenesis inhibitory effects[374] of ergosterol peroxide have also been reported.

In addition to ergosterol peroxide, a number of other steroidal endoperoxides have been reported, which are most commonly 5*α*,8*α*-epidioxysterols with variations in the side chains. A 5*α*,8*α*-epidioxysterol sulfate **592** was isolated from the cultured diatom *Odontella aurita*.[375] Four steroidal saponins, pariposides A–D **593–596**, were isolated from the roots of *Paris polyphyllava*. These compounds are the first spirostanol saponins with a peroxy group located between C-5 and C-8 of the aglycon.[376] Bioassay-guided fractionation of an

**570** $R_1 = I, R_2 = H, n = 8$
**571** $R_1 = I, R_2 = H, n = 6$
**572** $R_1 = H, R_2 = H, n = 8$

**573**

**574**

**575**

**576**

**579**

**577** R = H
**578** R = OH

**580** R =

**581** R =

**582**

extract of a marine sponge, *Lendenfeldia chondrodes*, has led to the isolation and identification of new epidioxy sterols **597** and **598** as an inseparable mixture, which might be formed in the sponge during sample storage and extraction.[377] A cytotoxic 5*α*,8*α*-epidioxysterol **599** was isolated from a soft coral *Sinularia* sp.[378] *Sinularia flexibilis* (Hainan Is., China) afforded two new members of the 5*α*,8*α*-epidioxygorgostane family of metabolites **600** and **601**, as well as the 22*α*,28-epidioxycholestane C-22 epimers **602** and **603**.[379] A group of 5*α*,8*α*-epidioxysterols, topsentisterols A1–A3 **604–606**, were isolated from a marine sponge *Topsentia* sp.[380] The marine sponge *Luffariella* cf. *variabilis* was the source for a series of 5*α*,8*α*-epidioxy sterols **607–609**.[381] *Eunicella cavolini* (Lichadonissia Is., Greece) provided another group of 5*α*,8*α*-epidioxysterols **610–612**.[382] Three epoxysteroids **613**, **614**[383] and **615**[384] were obtained from *Helianthus tuberosus* and *Lentinus edodes*, respectively. An undescribed endophytic *Phomopsis* species from *Maytenus hookeri* provided a new sterol **616**.[385] A peroxy steroid, 9(11)-dehydroxyaxinysterol **617**, from an Okinawan species of the genus *Axinyssa*, was found to inhibit the growth of several human cancer cell lines.[386] Fermentation of a *Rhizopus* sp. from the bryozoan *Bugula* sp. (Jiaozhou Bay, China) yielded a cytotoxic ergosterol **618**.[387]

In addition, several rare 5*α*,9*α*-epidioxy steroids have also been characterized. The mushroom *Pleurotus eryngii* afforded the first example of a naturally occurring 5*α*,9*α*-epidioxy-8*α*,14*α*-epoxy-6-ene sterol **619**.[388] One osteoclast-forming suppressing sterol, gargalol B **620**, was obtained from the mushroom *Grifola gargal*.[389] The mushroom *Lepista nuda* was the source for two new sterols, **621** and its C-6 epimer **622**.[390] Another two compounds of this class, **623** and **624**, were isolated from the mushroom *Hypsizigus marmoreus*.[384]

Besides endoperoxides, steroids containing hydroperoxy groups have also been identified. Two isomeric hydroperoxides, **625** and **626**, previously reported as synthetic products, were isolated as mildly cytotoxic metabolites of a Taiwanese collection of *Eudistoma* sp.[391] Three cytotoxic oxygenated fucosterols **627–629** were obtained from the marine brown alga *Turbinaria conoides*.[392] A Formosan soft coral *sinularia* sp. was the source for 7*β*-hydroperoxy-24-methylenecholesterol **630**, which exhibited significant cytotoxicity against P-388 tumor cell with a $ED_{50}$ of 2.6 μg/mL.[393] A pair of allylic regioisomers, **631** and **632**, were found from the bark of *Melia azedarach*, which showed significant cytotoxic effects against several human cancer cell lines.[394] A pregnane-

**583**

**584**

**585**

**586**

**587**

**588**

**589**

**590**

**591**

**592**

**593** $R_1$ = S1, $R_2$ = H
**594** $R_1$ = S2, $R_2$ = H
**595** $R_1$ = S3, $R_2$ = H
**596** $R_1$ = S1, $R_2$ =OH

S1

S2

S3

**597**

**598**

**599**

**600**

**601**

**602** 22*S*
**603** 22*R*

**604**

**605**

type steroid sclerosteroid E **633** was a constituent of the soft coral *Scleronephthya gracillimum*.[395] A chemical investigation of the roots of *Cynanchum stauntonii* has resulted in the characterization of a new hydroperoxide with a 13,14:14,15-disecopregnane-type skeleton, named stauntonine **634**, whose relative stereochemistry was determined by X-ray crystallographic diffraction analysis. The compound showed dose-dependent relaxation on aortic rings with endothelium contracted by phenylepherine or KCl.[396]

The structures of a series of peroxy function containing pregnane glycosides including periperoxides A–E **635–639**[397] and previously reported periplocosides A–K[398,399] have been revised to be orthoester group bearing ones using 2D NMR techniques as well as chemical transformations and X-ray crystallographic diffraction analysis.[400]

## 5 Fatty Acid Metabolites

Lipoxygenase (LOX) pathways are involved in the

**606**

**607** R = **608** R = **609** R = **610** R = **611** R = **612** R = **613** R = **614** R = **615** R =

**616**

**617**

**618**

**619**

**620**

**621** R = α-OH, β-H
**622** R = β-OH, α-H

**623**

**624**

**625** R = β-OOH
**626** R = α-OOH

**627**

**628**

production of important signal and defensive metabolites in mammals, higher plants, and algae.[401–404] In these pathways molecular oxygen is introduced into a polyunsaturated fatty acid to form an intermediate hydroperoxide, which may then be cleaved to give shorter chain-length oxygenated products, collectively known as oxylipins. Interestingly, different principles of transformations have been identified. While plants use almost exclusively C18 fatty acids for the production of oxylipins,[401] algae and animals rely predominantly on the transformation of C20 fatty acids.[403] In animals cleavage of the intermediate hydroperoxy fatty acids is achieved by a dual function of LOXes, while plants and algae rely often on hydroperoxide lyases (HPLs) to produce shorter chain oxylipins.[401–403]

The mechanism of fatty acid transformation in the Diatom *Thalassiosira rotula* does not, however, follow established lipoxygenase/hydroperoxide lyase pathways known from higher plants or mammals but rather relies on a unique transformation of polyunsaturated hydroperoxy fatty acids. These intermediates are then transformed to polyunsaturated short chain aldehydes and short chain hydroxylated fatty acids, which are novel oxylipins.[404] The similar transformation mechanism of fatty acid hydroperoxides has also been reported

**629**

**630**

**631**

**632**

**633**

**634**

from the moss *Physcomitrella patens*. The moss produces metabolites typical for animals, plants, algae, and mushrooms by new transformations of arachidonic acid, combining in a unique way metabolic themes from all these organisms.[405] Recent genome sequences leading to an increasing number of enzyme-mechanistic and structural analysis of LOXs and new members of the oxylipin pathway, as well as oxylipin profiling shed new light on the biosynthesis and occurrence of oxylipins in non-mammalian organisms. A review of these new aspects has been published.[406]

## 6 Conclusions

This article reviewed several hundreds of new peroxy natural products produced by terrestrial fungi, higher plants, and marine organisms not only their structures and chemistry, but also their diverse biological activities. However, only a limited number of them have been further evaluated since a limited supply of the active ingredients from the natural sources. It needs more research attention on total synthesis of important compounds and further biological evaluation. Further studies on their previously untapped resources with further unprecedented bioactive metabolites needs to be conducted. This review also emphasizes the role of peroxides from terrestrial fungi, higher plants, and marine organisms as an important source of leads for drug discovery.

## 7 Acknowledgements

This study was supported by the National Natural Science Foundation of China (NSFC 31100012, U1132607) and the National Basic Research Program of China (973 Program, 2009CB522300).

**Open Access** This article is distributed under the terms of the Creative Commons Attribution License which permits any use, distribution, and reproduction in any medium, provided the original author(s) and source are credited.
